# Integration of family planning into HIV services: a systematic review

**DOI:** 10.1080/07853890.2021.2020893

**Published:** 2022-01-31

**Authors:** Luka Nkhoma, Doreen Chilolo Sitali, Joseph Mumba Zulu

**Affiliations:** a School of Public Health, University of Zambia, Lusaka, Zambia; b Institute of Distance Education, University of Zambia, Lusaka, Zambia

**Keywords:** Family planning, HIV services, integration, health systems barriers and facilitators

## Abstract

**Background:**

The unmet need for safe and effective contraception still remains high. In 2017, about 25% of women of childbearing age who wanted to avoid pregnancy in the developing world were not using a modern contraceptive method. The biggest proportion (21%) of these women live in Sub-Saharan Africa. Little attention has been paid to the health systems factors impacting the integration of family planning into HIV services. This systematic review intends to document health systems factors constraining or facilitating the integration of family planning into HIV services.

**Methods:**

A search of electronic databases such as PubMed and Google Scholar was conducted using keywords. We considered peer-reviewed articles which were published in English between 1^st^ January 2010 and 31^st^ December 2020. The peer-reviewed articles which were considered focussed on identifying barriers and facilitators at the levels of the health system which influence the success or failure of integrated family planning and HIV programs, availability of integrated family planning services in HIV care, the evidence on the feasibility, effectiveness and cost-effectiveness of integrating family planning and HIV services and investigating the outcomes of programs aimed at strengthening family planning integration in HIV counselling, testing and care. Twenty-seven articles that identify factors affecting integration of family planning into HIV services met the inclusion criteria and were thematically analysed.

**Results:**

Health systems factors constraining integration of family planning and HIV services were human resource turnover and shortages, lack of policy guidance on integrated care, poor oversight, unclear service delivery guidelines, inadequate infrastructure and insufficient monitoring systems. Facilitators to the successful integration of family planning into HIV services were identified as training in family planning for service providers, the creation of a supportive policy environment to accommodate service integration, supportive supervision and a positive attitude by service providers towards service integration.

**Conclusion:**

Increase in the health workforce to support integrated service delivery, skills enhancement for service providers and improvement in family planning commodity stock levels play a key role in facilitating the integration of family planning into HIV services.

## PLAIN ENGLISH SUMMARY

The findings of this systematic review support integration of family planning into HIV services. The results indicate that it is both possible to integrate family planning into HIV services as well as to implement an integrated model to health care service delivery. Further, the review indicates that there are a number of health systems factors that facilitate as well as constrain the integration of the two services.

Integrating family planning into HIV services addresses a number of Sexual and Reproductive Health challenges being faced by women of childbearing age. Integrated family planning and HIV services reduce high rates of unintended pregnancies among HIV-positive women, reduce high pregnancy-related maternal mortality rates and increase the chance of meeting international and national development goals and targets, particularly Sustainable Development Goal number 3. Integration also helps women receive several services during a single visit to a health facility.

Integrated family planning and HIV services ought to support the provision of comprehensive family planning and HIV services in a country. This is critical to encourage especially women living with HIV infection to access a full range of contraceptive methods. The provision of comprehensive family planning and HIV services allows HIV negative as well as HIV positive couples and individuals to meet their sexual and reproductive health needs.

The review produced sufficient knowledge on the health systems factors which facilitate and constrain the integration of family planning into HIV services.

The systematic review demonstrated the need to deal with health systems barriers in order to ensure success in integrating family planning and HIV services. The review also demonstrated the need for health systems strengthening in order to provide quality integrated family planning and HIV services.

**Key Messages**
- Integration of family planning into HIV services is more and more being used as an approach for meeting the contraceptive needs of HIV-positive women of the childbearing age group.
- Training for service providers, supportive supervision, a supportive policy environment and a positive attitude by service providers towards integration were identified as health systems factors facilitating to the integration of family planning and HIV services.
- Staff turnover and shortage, inadequate infrastructure, stock-outs of family planning commodities, lack of coordinated leadership for integration, lack of integrated national policies and operational frameworks and separate funding for family planning and HIV services were identified as health systems barriers to integration of family planning and HIV services.

## Background

The unmet need for contraception continues to be a global public health problem among HIV-negative and HIV-positive women of childbearing age [1]. In 2017, about 214 million women aged 15–49 years reported an unmet need for contraception [2]. Most of the women with unmet need for contraception live in underdeveloped regions of the world [3]. Among all the regions of the world, sub-Saharan Africa has the highest rate (21%) of unmet need for contraception [4]. The unmet need for contraception is greater in rural areas compared to urban areas [5].

Above 80% of unintended pregnancies among HIV-negative and HIV-positive women globally are as a result of an unmet need for contraception [6]. In Sub-Saharan Africa, about 55.9% of pregnancies among women living with HIV are unintended [7]. Unintended pregnancies among HIV-positive women are associated with a high maternal mortality rate which is ten times more than that of women who are HIV-negative [8]. Integrating family planning and HIV services is thus necessary to reduce high rates of unintended pregnancies, high pregnancy-associated maternal mortality rates and to be able to attain international and national development goals and targets, especially the Sustainable Development Goal 3 [4].

Integration has been defined in many ways [9]. From a recipient point of view, integration is concerned with health care that is easy to navigate [9]. It is a service that is well harmonised and reduces the number of stages in an appointment and the number of separate visits required to a health facility [9]. From the health systems perspective, integration takes place when decisions on policies, financing, regulation and delivery are properly sorted [9].

Providing family planning and HIV services jointly is essential to guaranteeing universal access to family planning services and HIV prevention, treatment, care and support services [10]. Integration also inspires efficient resource utilisation because of better administration of available resources [11]. In addition, it lessens the repetition of actions and leads to shorter waiting times [11]. Further, integration may be used as a tool for generating concerted effort in dealing with lost chances in HIV prevention and family planning services at all service delivery levels [4]. Another benefit of integrating family planning and HIV services is that it can be used as a catalyst for the improvement of the quality of health care services delivered and for increasing the levels of client satisfaction [4]. Integration also encourages the choice of services centred on the full needs of individual patients [10]. Reviews conducted have mainly focussed on the evidence of the feasibility, effectiveness and cost-effectiveness of integrating family planning into HIV services and the range of models used to integrate the two programs [12,13].

## Methods

### Search strategy

Studies that investigated the integration of family planning into HIV services were identified by searching eighty-eight databases. The following terms were entered into PubMed and Google Scholar “FP” [tab] or “integration” [tab] or “HIV” [tab] or “services” [tab] or “comprehensive” [tab] or “facilitators” [tab] or “barriers” [tab] or “health” [tab] or “factors” [tab] and “systems” [tab]. To find reports on integration, we examined a number of electronic databases through the use of suitable keywords. The search was restricted to the English language.

### Inclusion criteria

**Figure 1.**
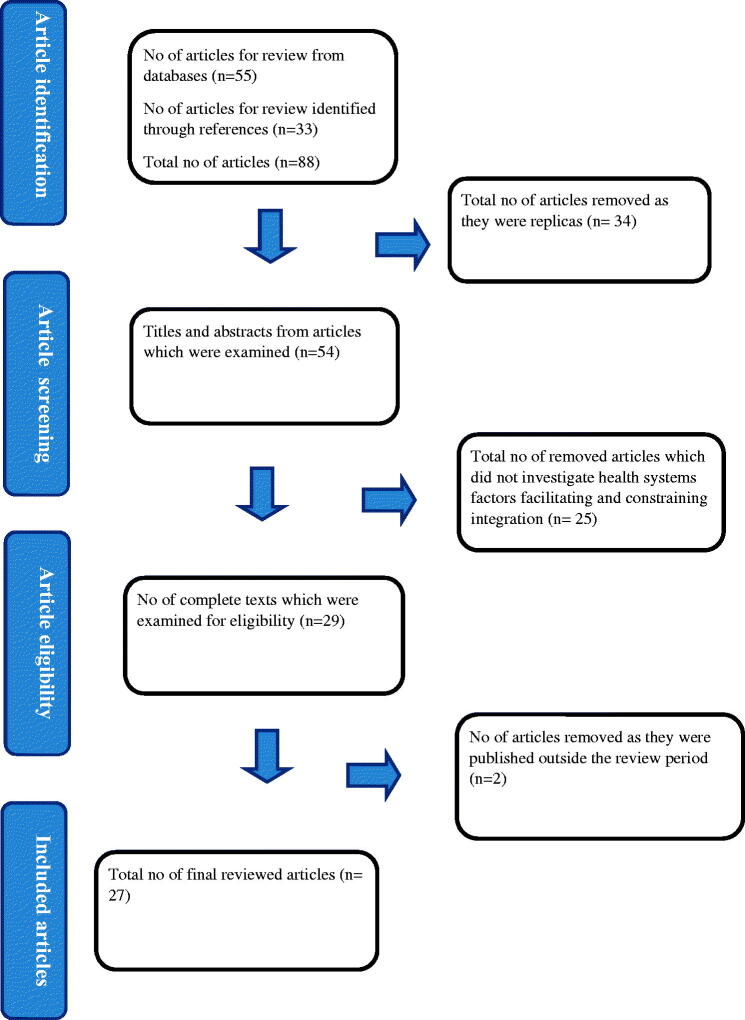
PRISMA flow diagram.

Studies met the inclusion criteria for the review if they were published in a peer‐reviewed journal between 1^st^ January 2010 and 31^st^ December 2020. Studies were also included if they provided data on the integration of family planning into HIV services. We included any model of family planning integration into HIV services in which the provision of family planning services took place at the family planning department, in the HIV department or through referrals to the family planning departments. The primary outcome of interest for the review was the health systems factors that may facilitate or constrain the integration of family planning into HIV services (Figure 1).

### Data analysis and synthesis

We analysed data from the chosen articles using the thematic analysis technique. Thematic analysis assisted us to find themes and associations from the data which was coded.

## Main results

Three of the integrated programs were evaluated in Zambia [13–15]. The remaining twenty-four integrated programs were evaluated in other African countries [4,16–30,31–36]. The majority of the studies integrated family planning with HIV testing (*n* = 18) or HIV treatment and care (*n* = 9). Integration of family planning and HIV was found to be possible and cost-effective. Service provider training, supportive supervision, creation of a supportive policy environment and a positive attitude of service providers towards integration were identified as enablers to the integration of family planning and HIV services. Shortage of human resources, inadequate infrastructure, family planning commodity stockouts, lack of coordinated leadership for integration, lack of integrated national policies and operational frameworks and separate financing for family planning and HIV programs were identified as barriers to integration of family planning and HIV services (Table 1).

**Table 1.** Characteristics of included studies.

Author/country Period	Study type	Sample characteristics Setting and area where integration was done	Study objectives	Study findings	Outcomes
1. Baumgartner et al., 2014 Tanzania/August, 2014	Cross-sectional study	Interviews with 300 CTC female clients in Iringa and Morogoro (Urban and Rural) areas	To examine the success of an aided referral approach of integrated family planning and HIV services in Tanzania	Low staff levels was identified as a constraint to integration	Facilitated referral model is a feasible
2. Chabikuli et al., 2009 Nigeria/November 2009	Retrospective survey	Reviews of registers at 4 tertiary hospitals, 60 secondary hospitals and 7 Primary Healthcare facilities between March 2007 and January, 2009 Urban and rural areas of Nigeria	To assess variations in service utilisation in an integrated family planning and HIV service model	Medical fee charges on family planning and HIV services was identified as a constraint	Improved contraceptive uptake
3. Mutemwa et al., 2017 Kenya/November, 2017.	Cross-sectional study	Observed 366 client-provider consultation sessions 37 interviews for service providers Central Province of Kenya	To examine the relationship between integration and technical quality in public health facilities	Insufficient family planning supplies, lack of infrastructure plus weak supervision were identified as constraints to integration	Better technical quality of care
4. Kanyangarara et al., 2019, Benin, Burkina Faso, DRC, Malawi, Senegal, Sierra Leone, Tanzania, Togo, Uganda and Zimbabwe/2012–2015	Cross-sectional facility-based survey	Secondary analysis of facility-level data in 10 Sub-Saharan Countries	To evaluate the accessibility of integrated family planning services and related factors in HIV supported facilities	The presence of family planning supplies was an enabler while the none-availability of guidelines and the absence of skilled staff was a constraint to integration	Shortcomings in the preparedness
5. Irani et al., 2015, Malawi/December, 2015	Facility-based assessment	Facility audits (*n* = 41), Interviews (*n* = 163), Client Exit Interviews (*n* = 425), Mystery client visits (*n* = 58) and Focussed group discussions (*n* = 3), December 2015 North, Central and Southern rural and Urban districts of Malawi	To establish the level of integration of family planning into HIV services and investigate system-level constraints to integration	Absence of skills development and a lack of enforcement of national guidelines were identified as constraints	High unmet need for contraception
6. Brunie et al., 2017 , Uganda/June, 2017	Two-arm cluster randomised controlled study	Interviewed 36 Village Health Teams and 256 family planning clients May 2012 to September 2013 Rural and Urban Uganda	To carry out an evaluation of a community-based approach in family planning and HIV service integration	Skills development and supervision were identified as enablers to integration	Integration is possible
7. Mutisya et al., 2019 Kenya/August to October, 2013	Cross-sectional study	Interviewed 100 service providers and 40 clients August to October 2013 Urban areas	To measure the level of integration of family planning, HIV plus other basic health care services in health facilities	Lack of family planning commodities was identified as a constraint while staff capacity and positive attitude were identified as enablers	Enhanced through targeted interventions at the facility
8. Silumbwe et al., 2018 Zambia/May 2019	Exploratory design	Twelve focus group discussions were conducted with community members (*n* = 114) and two with healthcare providers (*n* = 19). Ten in-depth interviews were held with key stakeholders Urban areas in Kabwe District	To investigate factors that facilitate and constrain family planning and contraceptive service delivery and use	None availability of policies and family planning supplies were identified as obstacles to integrated care	Community and health systems factors
9. Kosgei et al., 2011 Kenya/December, 2011	Retrospective Cohort Study	Review of patient data records	To establish the effect of family planning and HIV service integration on contraceptive methods utilisation and numbers of pregnancies	Donor restrictions in the use of funds was an obstacle to service integration	Increased use of modern FP methods
10. Zewdie et al., 2020 Ethiopia /December 2017 to April, 2018	Cross-sectional study	Interviews with 517 HIV positive women Urban Ethiopia	To assess the level of family planning integration into HIV services	Low human resource numbers, lack of skills development and irregular family planning supplies were identified as constraints to integration	Women were not using modern contraceptive No method -mix at HIV clinics
11. Steinfeld et al., 2013 /Kenya May and June 2010	Cluster randomised controlled trial	Thirty open-ended interviews with HIV positive men, rural Kenya	To investigate the barriers and enablers to the utilisation of family planning among HIV positive men	Staff shortage was identified as a constraint to integration	Preferred way of receiving family planning services
12. Faye et al., 2017 /Zambia /October 2013 to September, 2014/	Cross-sectional, non-randomised comparison design	900 reviews of medical records 20 interviews with facility in-charges 150 patient exit interviews Urban areas of Zambia	To approximate the expenditures and match the cost-efficiency of integrated models	Absence of human resources was identified as a constraint while the presence of policy guidelines, skills development and supportive supervision were identified as facilitators to integration	Integrated model of family planning and ART services are not necessarily more cost-effective
13. Close et al., 2012 Malawi and Tanzania /2014–2015	Retrospective cross-sectional study	Facility inventory questionnaire, interviews with facility providers, observations and exit interviews with clients, urban and rural Malawi and Tanzania	To evaluate the accessibility and quality of essential primary health services across health systems	Availability of family planning commodities was identified as a facilitator to integration	HIV integration is associated with FP quality of care.
14. Bintabara et al., 2017 /Tanzania October 2014 and February 2015	Survey	1188 participants Facility inventory, interviews, observations at ANC and exit interviews with FP clients	To measure the level and establish the contributing factors of family planning uptake and HIV testing	Nonexistence of policy guidelines and training were identified as constraints to integration	Unsatisfactory readiness
15. Mak et al., 2013 , 2009, Kenya and Swaziland/2009	Three-stage clustered Surveys	1,632 interviews conducted in urban areas of Swaziland and Kenya	To establish the degree of need for family planning and HIV prevention services and investigate service utilisation forms	Insufficient human resource and the absence of training was identified as a barrier to integration	Demand creation at the community level and provider-initiated integrated service provision
16. Chibwesha et al., 2011, /Zambia/April, 2011	Cohort study	18,407 interviews with HIV positive women	To measure the utilisation of double family planning method-mix in the prevention of unwanted pregnancies	Undesirable service provider attitude was seen to be a constraint to integration	Feasibility of integrating comprehensive reproductive health counselling into HIV care and treatment
17. Wanyenze et al., 2015, Uganda/February-June, 2011	Cross-sectional study	797 interviews with HIV-infected women	To evaluate fertility needs and factors influencing the unmet need for family planning in HIV positive women	Lack of family planning commodities was identified as a constraint to integration while provider skill was identified as a facilitator to integration	High unmet need for family planning among HIV infected women
18. Tweya et al., 2017 /Malawi/ April 2012 and March 2013	Retrospective-longitudinal Cohort	Analysed 20,253 HIV-positive women’s records February 20122 to December, 2016	To examine patterns in contraceptive method utilisation post-Electronic Medical Record System	Low human resource levels with high patient numbers in ART units was a constraint to integration	Contraceptive use increased
19. Grossman et al., 2013 /Kenya/December, 2009 to September, 2011	Cluster-randomised trial	5,682 clinical encounters from baseline period (December 2009–February 2010) and 12,531 encounters from end-line period (July 2011–September 2011, 1 year after site training). Kenya	To establish if family planning and HIV integration is related to increased utilisation of more effective family planning methods	Harmonised leadership was identified as an enabler to integration	Increased use of more effective contraceptive methods
20. Wall et al., 2018, Rwanda/ April 2014 and June 2014	Qualitative case study	Interviews with 10 Policy makers	To examine the awareness levels, attitudes and practices among policy makers and stakeholders who manage integrated family planning and HIV services	Staff training was identified as an enabler in integrated family planning and HIV services	Improved FP method knowledge
21. Newmann et al., 2013 Kenya/ November 2007 and October 2008	Mixed-method study	Interviews 31 providers, November 2007 – October 2008	To provide up to date information on interventions on family planning and HIV care services	Insufficient numbers of human resources and infrastructure were identified as constraints to integration	Unmet need for contraception and unintended pregnancy
22. Harrington et al., 2012 Kenya/July and September 2009	Qualitative case study	Thirty open-ended interviews were conducted with HIV positive women July – September 2009	To establish the fertility plans and family planning choices among HIV positive women	Irregular family planning supplies was identified as an obstacle to integration	Acceptability of integrated FP and HIV services
23. Hawkins et al., 2020 Botswana June 2018 to August 2018	prospective, hybrid type 2 clinical intervention and implementation study	141 women completed the survey, and 107 did so post-intervention October 2017 to March 2018	To evaluate a health facility approach for addressing unmet need for contraception	Absence of training and skills among service providers was identified as a constraint to integration	Family planning discussions and increased interest in LARC.
24. Awadihi et al., 2012 Tanzania/December, 2012	Mixed method study design	Questionnaires were administered to 147 randomly selected service users and 35 health providers while 10 in-depth interviews were conducted among Ministry of Health and Local government. Four focus group discussions were conducted among HIV voluntary counselling and testing (VCT) service users Urban Tanzania	To measure the ability to integrate family planning and HIV services and testing	The absence of policy guidelines and protocols for integrated services and low staff motivation levels were barriers to integration	Integration of FP and HTC is feasible and acceptable
25. Nattabi et al., 2011 Uganda February and May 2009	Mixed method design	Interviews with 476 PLHIV, rural and urban Uganda	To investigate skills, accessibility and determinants of family planning utilisation among HIV positive people	Lack of training was identified as a constraint to integration while the regular supply of family planning commodities was identified as a facilitator to integration	Overcome individual and structural barriers
26. Makonnen et al., 2020 Ethiopia/ June 2015 to November 2018	Cross-sectional design	Interviews with 403 clients and 305 service providers in urban areas of Ethiopia	To identify the advantages and disadvantages of integrating family planning and HIV services	Availability of sufficient and skilled health workforce and were identified as enablers to integration	Offering an integrated service at a one-stop facility by far outweighing the disadvantages
27. Hope et al., 2014 Kenya/ December, 2014, Nigeria, Rwanda, Mozambique and Tanzania	Scoping study	examined the literature on national and international strategies to integrate SRH and HIV services using a scoping study methodology, urban area of Kenya, Nigeria, Rwanda, Tanzania and Mozambique	To gather evidence on strategies for integrated sexual and reproductive health and HIV services	Lack of leadership and governance, integrated policies, independent financing and lack of training were identified as constraints to integration	Delayed or incomplete integration of higher-level health systems functions:

## Health systems factors facilitating and constraining integration of family planning into HIV services

### Factors facilitating integration

#### Training for service providers

Formalised training of service providers in insertions and removals of Long-Acting Reversible Contraceptives (LARC) was identified as key to the success of the provision of integrated family planning services among women who are HIV positive in health facilities [37]. Training included formalised classroom-based with certification, through workshops and through ongoing practical sessions through supportive supervision within hospitals and clinics by family planning Specialists [37]. Training facilitates integration by equipping service providers with the required competencies to provide quality integrated family planning and HIV services. Training also improves the skills in clinical practice as well as in professional competencies and this, in turn, ensures success in service integration.

#### Supportive supervision

Supportive supervision was also identified as an enabler in the integration of family planning and HIV services [38]. Repetitive checks, corrective support and mentorship were identified to be critical in facilitating knowledge gain about the integration process and improving the clinical practice by service providers [21]. Most on-the-job mentorship programs took place within hospitals and clinics during supportive supervisory visits [37]. Regular supportive supervision helped supervisors to promptly identify the targets and determine if these targets will be met, thereby holding service providers responsible for full implementation of the family planning and HIV interventions [21].

#### Supportive policy environment

A supportive policy environment with clear policy guidelines was identified as an enabler in the integration of family planning and HIV services [39]. A safe and supportive environment with clear policy guidelines improves the willingness of service providers to support and deliver integrated family planning and HIV services [39]. The Clinical Management of HIV in Children and Adults Policy in Malawi recommends that providers should offer all HIV positive clients with condoms, injectable family planning methods for female clients and refer clients to another provider or site if clients prefer another family planning method. The UNAIDS policy on HIV Testing and Counselling (1997) recommends an increase in women’s voluntary access to VCT services and those women should be offered information on reproductive health and infant feeding. The Uganda policy on Voluntary Counselling and Testing recommends that Counsellors should assess family planning needs for women and refer them to service providers.

#### A positive attitude by service providers towards integration

A positive attitude by service providers towards integration was also identified as a facilitator in the integration of family planning and HIV services [4]. A positive attitude creates a sense of ownership of the integration process, helps service providers to understand without bias what ought to be integrated and how to integrate the two services [4]. A sense of ownership of the integration process is an example of the positive attitude towards integration by service providers [4]. Another positive attitude towards integration is the providers’ desire to help HIV-infected women make informed choices about birth spacing and limiting [40].

### Factors constraining integration

#### Human resource turnover and shortage

Inadequate numbers of human resources for health at HIV clinics were identified as a barrier to the integration of family planning into HIV services [16]. For the client, human resource shortage is a barrier because it results in increased waiting time and delayed attention. For service providers, shortage of human resource increase workload on a few available staff leads to burnout and compromises the capacity of the already overburdened health personnel to meet service delivery requirements [41]

#### Inadequate infrastructure

The absence of appropriate physical space in public health facilities was identified as another barrier to the integration of family planning and HIV services [42]. No health care service can successfully be provided without the availability of basic infrastructure [43]. The absence of physical space makes it difficult to maintain privacy and confidentiality for family planning and HIV clients [39]. Lack of infrastructure also affects the process of physical co-location of family planning and HIV services [39].

#### Stock outs of family planning commodities

Family planning commodity stockouts were identified to have negative consequences in integrated health care service delivery and were, therefore, a barrier to integration [4]. For the client, family planning stockouts represent an access barrier and create anxiety and uncertainty in women because of fear of unwanted pregnancy [14,44]. For service providers, stockouts of family planning commodities affect the skill levels as they are unable to acquire or maintain skills to provide certain family planning methods, especially the long-acting and reversible contraceptive methods due to lack of practice [44]. At the facility level, family planning stockouts affects the success of health facilities in achieving their set targets [44]

#### Lack of coordinated leadership for integration

Lack of coordinated leadership is an impediment to the integration of family planning and HIV services [41]. Uncoordinated leadership inhibits coordinated planning for integrated services, promotes program territorialism and raises budgetary concerns [41].

#### Lack of integrated national policies and operational frameworks

Lack of integrated national policies was also identified as a barrier to the integration of family planning and HIV services. Integrated policies on integration are important because they bring together decisions and support functions across different parts of the health care service [9]. Integrated policies and operational frameworks support the development of appropriate care systems, processes and quality standards [45]. Further, integrated policies and national operational frameworks support the holistic evaluation of integrated systems and programs [45]. The absence of integrated national policies and guidelines on the other hand results in fragmentation in service delivery as well as creating boundaries in health care service delivery [45].

#### Separate financing for family planning and HIV services

Separate financing for family planning and HIV services was another barrier identified to integration. Separate financing undermines progress towards service integration because it affects health system quality and efficiency by compromising budgetary and planning, misalignment of incentives, and duplication and miss-targeting of services [46]. Separate financing may also lead to increased administrative costs and reduced bargaining power for purchasing thereby negatively impacting the integration process [46].

## Discussion

The twenty-seven studies reviewed investigated barriers and facilitators to integration of family planning and HIV testing, care and treatment services. The reviewed studies showed that integration of family planning into HIV services is achievable. The studies also showed that there are a number of health systems factors that can facilitate and constrain the integration of family planning into HIV services.

Human resources for health were identified as a barrier to the success of the integration of family planning and HIV services [16]. The capacity of a health system to integrate family planning and HIV services mainly hinges on the placement, enthusiasm, skills and knowledge of the health workforce. This is because a country’s health workforce is the one that is in charge of organising and providing integrated health care services. To achieve success in integration, there ought to be sufficient numbers of health workers operating in the family planning and HIV departments and these health workers ought to have the right mix of skill, knowledge and motivation. It is therefore important that before integrating health care services, the recruitment, distribution and retention processes are evaluated and strengthened. To improve skills and knowledge on integration, pre-service and in-service training for service providers may need to be conducted while incentives may also need to be provided in order to improve staff motivation.

Separate financing for family planning and HIV services were also identified as an impediment to the integration of family planning and HIV services [46]. Finances are used to support health systems to procure family planning and HIV medical supplies, recruit and pay emoluments for health personnel and are also used to support health promotion and preventive activities. Financing family planning and HIV services separately undermine the progress towards service integration because it affects health system quality and efficiency by compromising budgetary and planning, misalignment of incentives, and duplication and miss-targeting of services. Separate financing may also lead to increased administrative costs and reduced bargaining power for purchasing thereby negatively impacting the integration process of family planning and HIV services.

Stock-outs of medical supplies, especially family planning commodities, is another health system factor identified as a barrier to the integration of family planning and HIV services [4]. To function well, a health system must ensure equitable access to essential medical products, including family planning commodities. Regular stock out of family planning commodities is a barrier not only to the attainment of universal access to health but also to the success of the integration of family planning and HIV services. In the absence of family planning commodities, an attempt to integrate family planning and HIV services may prove to be difficult.

Leadership and governance were also identified as an impediment to the integration of family planning and HIV services [39]. Leadership and governance guarantee that a strategic policy framework for service integration is in place and mechanisms for conducting regular supervisory visits are also in place. The absence of clear policy and service delivery guidelines affects effective monitoring of integrated services and coalition-building. A safe and supportive policy environment with clear policy guidelines on the other hand improves the willingness of service providers to support and deliver integrated family planning and HIV services. Clear policy guidelines at various health provision levels, therefore, assure efficiency in integrated service delivery.

Good service delivery is a key ingredient of any health system [47]. Service delivery guarantees access and coverage to integrated family planning and HIV services. In a well-integrated health system, services are of high quality, people-centered and are well coordinated.

## Limitations of the review

This review has some limitations. One of the limitations is that although an extensive search and screening process was carried out, we may not have identified all suitable studies. Another limitation arises from the fact that the current evidence-based on health systems factors facilitating or constraining integration is scant.

## Conclusions

The results of this review indicate that it is both possible to integrate family planning into HIV services as well as to implement the integrated approach to health care service delivery. The results also show that with skill’s development for service providers, consistency in family planning commodity supply and the presence of appropriate physical infrastructure for integrated service delivery, integration of family planning into HIV services can be achieved and can lead to improved health outcomes. However, there is still a need to conduct further studies to investigate and analyse other health systems factors which act as facilitators or barriers to integration. Factors such as leadership and governance, financing, health information systems, health workforce and service delivery can affect the integration of health care services and ought to be analysed.

## Data Availability

Data sharing is not applicable to this article as no datasets were generated during the current study.

## Author’s information

LN is a holder of a Master of Public Health Degree and is currently studying for a PhD in Public Health at The University of Zambia.

## Abbreviations

HIV: Human Immunodeficiency Virus
WHO: World Health Organisation
LARC: Long Acting Reversible Contraceptives
USAID: United States Agency for International Development
FP: Family Planning
UNFPA: United Nations Population Fund Agency
